# In Silico Genome Analysis Reveals the Evolution and Potential Impact of SARS-CoV-2 Omicron Structural Changes on Host Immune Evasion and Antiviral Therapeutics

**DOI:** 10.3390/v14112461

**Published:** 2022-11-06

**Authors:** Dhruv Chauhan, Nikhil Chakravarty, Arjit Vijey Jeyachandran, Akshaya Jayakarunakaran, Sanjeev Sinha, Rakesh Mishra, Vaithilingaraja Arumugaswami, Arunachalam Ramaiah

**Affiliations:** 1 Tata Institute for Genetics and Society, Centre at inStem, Bangalore 560065, India; 2 Department of Epidemiology, University of California, Los Angeles, CA 90095, USA; 3 Department of Molecular and Medical Pharmacology, University of California, Los Angeles, CA 90095, USA; 4 Department of Molecular, Cell and Developmental Biology, University of California, Los Angeles, CA 90095, USA; 5 All India Institute of Medical Sciences, New Delhi 110029, India; 6 Eli and Edythe Broad Center of Regenerative Medicine and Stem Cell Research, University of California, Los Angeles, CA 90095, USA

**Keywords:** SARS-CoV-2, Omicron, COVID-19, evolution, receptor binding domain, T-cell epitope, spike protein, drug

## Abstract

New variants of SARS-CoV-2 continue to evolve. The novel SARS-CoV-2 variant of concern (VOC) B.1.1.529 (Omicron) was particularly menacing due to the presence of numerous consequential mutations. In this study, we reviewed about 12 million SARS-CoV-2 genomic and associated metadata using extensive bioinformatic approaches to understand how evolutionary and mutational changes affect Omicron variant properties. Subsampled global data based analysis of molecular clock in the phylogenetic tree showed 29.56 substitutions per year as the evolutionary rate of five VOCs. We observed extensive mutational changes in the spike structural protein of the Omicron variant. A total of 20% of 7230 amino acid and structural changes exclusive to Omicron’s spike protein were detected in the receptor binding domain (RBD), suggesting differential selection pressures exerted during evolution. Analyzing key drug targets revealed mutation-derived differential binding affinities between Delta and Omicron variants. Nine single-RBD substitutions were detected within the binding site of approved therapeutic monoclonal antibodies. T-cell epitope prediction revealed eight immunologically important functional hotspots in three conserved non-structural proteins. A universal vaccine based on these regions may likely protect against all these SARS-CoV-2 variants. We observed key structural changes in the spike protein, which decreased binding affinities, indicating that these changes may help the virus escape host cellular immunity. These findings emphasize the need for continuous genomic surveillance of SARS-CoV-2 to better understand how novel mutations may impact viral spread and disease outcome.

## 1. Introduction

Severe acute respiratory syndrome coronavirus 2 (SARS-CoV-2) is a positive-sense, single-stranded RNA virus with approximately 30 kilobases of genome coding for 29 proteins from 15 open reading frames. SARS-CoV-2, which shows zoonotic origination from bats, was first detected in Wuhan, China in 2019 [1,2,3]. The causative virus of coronavirus disease 2019 (COVID-19), SARS-CoV-2 has infected over 596 million people worldwide, prompting a massive human health crisis [4]. As the pandemic progresses, new variants of SARS-CoV-2 continue to emerge with amplified virulence or transmission rates. Host immunological responses, viral adaptation to human hosts, lack of global vaccine coverage, and failure to follow the preventive procedures (e.g., wearing a well-fitting mask, keeping hands clean, and maintaining physical distance) have contributed to the continuous emergence of novel SARS-CoV-2 variants. To date, Alpha, Beta, Gamma, Delta, and Omicron variants of concern (VOCs) have been identified. Like other RNA viruses, SARS-CoV-2 has been continuously evolving through genetic mutations or viral recombination and structural changes, including insertions and deletions in different viral genes. These genetic changes have impacted physical and biological properties of SARS-CoV-2, resulting in increased viral spread, reinfection rates, and clinical severity, as reported among recent VOCs.

Lineages of SARS-CoV-2, such as B.1.1.7 (Alpha variant) in the United Kingdom, B.1.351 (Beta variant) in South Africa, P.1 (Gamma variant) in Brazil, and B.1.617.2 (Delta variant) in India, were purported based on mutations at various genes [5,6,7,8,9,10,11,12]. The Delta variant is responsible for more infections and spreads faster than the previously reported Alpha and Beta VOCs, thus attaining much international attention [13]. On 26 November 2021, the World Health Organization (WHO) designated the novel variant B.1.1.529 (Omicron variant), initially reported from South Africa, as a VOC based on the evidence that several mutations, particularly in the Spike protein, may have an impact on viral behavior such as transmissibility, infectivity, clinical severity, risk of reinfection, and the potential impact on diagnostics, prevention, and treatments [13]. Initial reports illustrated that Omicron has increased potential for faster viral spread than Delta and reinfection of fully vaccinated, boosted, and previously infected individuals [14]. The Omicron variant has also shown increased potential to evade pre-existing antibodies, T-cell immunity, and overall human immune action [14]. Thus, this variant has become more prevalent worldwide, overtaking Delta and other VOCs and quickly spreading to all continents [15]. With the Omicron variant being the leading cause of mortality and morbidity in the current phase of the COVID-19 pandemic, we analyzed the genome of VOCs, including the Omicron variant, with the aim of gaining insights into geographical distributions, evolutionary relationships, and the potential impact of key mutations identified in Omicron variants in altering therapeutics and immunity.

## 2. Materials and Methods

### 2.1. SARS-CoV-2 Data Analyses

The metadata and genomic and amino acid sequences of SARS-CoV-2 were retrieved from the *Global Initiative on Sharing All Influenza Database* (GISAID) (https://www.gisaid.org/, accessed on 10 October 2022). The VOC metadata was used to analyze the distribution of SARS-CoV-2 infection among different age groups and genders globally and those specific to the Omicron variant. All existing SARS-CoV-2 sequences submitted (6,104,697 Omicron sequences and 5,897,516 sequences for other VOCs) in the GISAID were utilized for this study. The number of VOC sequences reported in GISAID across the world was shown on a world map using packages geojsonio v0.10.0 and ggplot2 v3.3.6 in R v3.6.3. A density plot was generated for comparing the probability distributions of five VOC sequences based on age using ggplot2 v3.3.6 in R v3.6.3. The mutation metadata were organized into groups with respect to VOCs, with non-VOC data filtered out for further analysis. VOC mutations were mapped to the four structural proteins: envelope (E), membrane (M), nucleocapsid (N) and spike (S) and non-structural proteins nsp1–16, ns3, ns6, ns7a, ns7b, and ns8. We compared the structural protein mutational landscape of the five VOCs (Alpha, Beta, Gamma, Delta, and Omicron) to identify unique and common mutations. Mutations in different functional domains of spike were further analyzed. To illustrate the mutations in the context of 3D protein structure, the protein’s tertiary monomeric structure for the reference Wuhan-strain spike protein was modelled based on the template structure (PDB ID: 6VXX) in the SWISS-MODEL server [16]. The PyMol (https://pymol.org/2/, accessed on 2 January 2022) program was used to visualize the protein 3D structures. For the phylogenetic analysis, the subset of globally circulating VOCs’ sequences, created by Nextstrain (accessed on 22 October 2022), was used (Table S1) [17]. The molecular clock tree option was used to visualize the phylogenetic tree.

### 2.2. SARS-CoV-2 Target Protein and Drug Interaction Analysis

A total of eight representative inhibitors—namely parecoxib, chlortetracycline, ivermectin B1a, ivermectin B1b, sulfasalazine, remdesivir, atovaquone, and nirmatrelvir (a component of Paxlovid (Pfizer))—were selected as described in the previous reports [18,19] for a molecular docking study. To investigate the impact of novel mutations in Omicron proteins on the potency of the above-mentioned inhibitors, we first modelled the spike structural protein and three non-structural proteins nsp3, nsp5, and nsp12 using known protein structures (PDB ID: 7CWN, 6Y2E, 6LU7, and 6M71, respectively) as a template in the SWISS-MODEL server. For additional comparison, we modelled the structures of these four proteins from a reference EPI_ISL_402124 and a representative Delta variant (EPI_ISL_1470937, EPI_ISL_4577393). Subsequently, these predicted structures were used for molecular docking with their respective drugs in AutoDock Vina v1.1.2 [20]. The drug-binding cavities in the protein structures were determined based on a previous study [21]. The interacting residues of the target protein and the nature of their interactions were identified using LigPlot+ v2.2.4 [22]. A lower binding affinity score indicates higher affinity of the drug to the given protein. Hence, we only considered the best docking model for each protein exhibiting the high binding affinity score to the drug, as they are more likely to bind and inhibit the virus.

### 2.3. Prediction of T-Cell Epitopes (TCEs) and Their Binding Affinity to Predominant HLA Alleles

To identify the potential impact of Omicron strain mutations in human host immunity, amino acid sequences for the nsp3, nsp5, nsp12, and spike proteins of the reference strain and two variants, i.e., Delta and Omicron, were used to predict the 15-mer CD4 TCEs using the *Immune Epitope Database* and Analysis Resource (IEDB) MHC-II binding prediction tool under the IEDB-recommended prediction method (v2.22) [23]. In order to identify the most immunodominant CD4 epitopes among the predicted potential 15 mer peptides from these four proteins, the binding affinity was predicted using the six most prevalent Human Leukocyte Antigen (HLA; MHC Class-II) alleles (http://allelefrequencies.net/, accessed on 8 January 2022) reported from global populations [24]. The epitope binding prediction results are given in units of IC 50 nM, where a lower number indicates high binding affinity. Thus, we considered the peptides having high binding affinity score (score IC ≤ 10 nM) as having the potential to bind to T-cell receptors and stimulate an effective adaptive T-cell immune response [2]. We also identified the mean binding affinity score (IC ≤ 10 nM) of the predicted peptides with HLA alleles of the global population to evaluate the generality of those predicted epitopes.

## 3. Results and Discussion

### 3.1. Geographical Distribution of Omicron Variant

**Figure 1:**
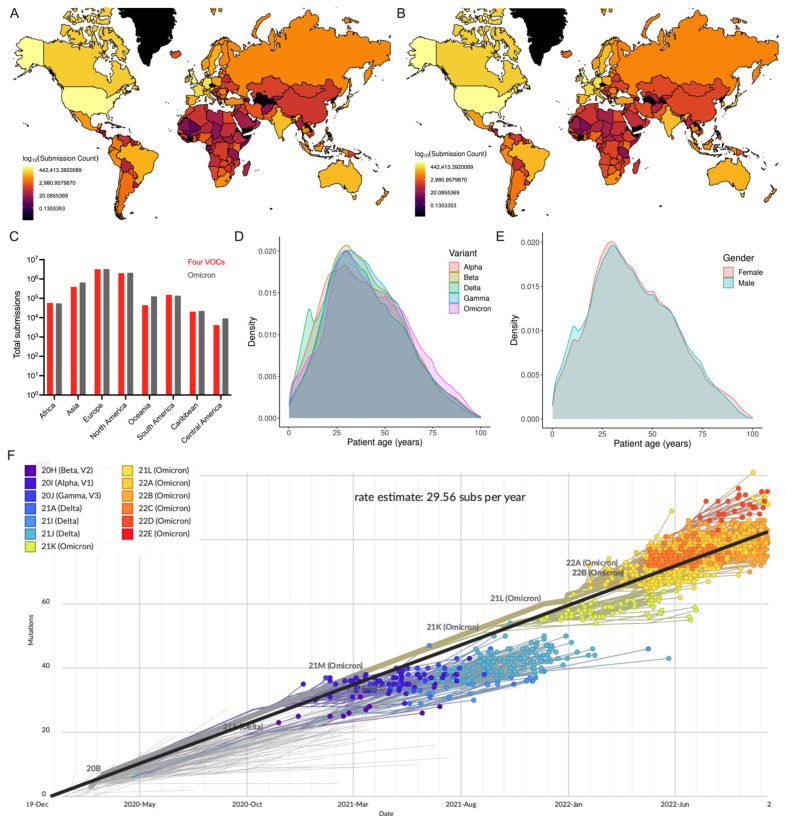
Global distribution of SARS-CoV-2 VOCs as reported in GISAID. (**A**) Number of Omicron and (**B**) all VOC genome sequences reported in GISAID. (**C**) Number of four VOCs and Omicron genome sequences (continent-wise) are presented. (**D**) Density plot shows the probability density distribution of five VOC genomic sequences based on age of COVID-19 cases. Only 38% (4.55 million) of the VOC sequences described the patient’s age (0–100 years) were considered. (**E**) Density plot displays the distribution of the consolidated number of all five VOC genomic sequences based on gender. (**F**) Phylogenetic tree based on a subset of globally circulating VOCs sequences created by Nextstrain (accessed on 22 October 2022).

The metadata analysis of 12,002,213 genome sequences (including Alpha: 1,192,900; Beta: 43,874; Gamma: 129,871; Delta: 4,530,871; Omicron: 6,104,697) revealed that a greater number of sequences were reported from Europe, followed by North America (Figure 1A–C). This sequence data also show that the number of Omicron sequences deposited in GISAID elevated the total number of the remaining four VOC sequences. The comparison of the distributions of five SARS-CoV-2 VOC sequences revealed that people within the age group of 26–35 years were predominantly infected with the Omicron variant, which is consistent with the overall pattern observed across SARS-CoV-2 VOC infections (Figure 1D). While 60% of all VOC sequences have no gender information, the demographic data from the remaining 4,796,586 (21.1% female; 18.9% male) sequences revealed that there is a propensity for females to be susceptible to COVID-19) (Figure 1E). This genomic epidemiological data shows a correlation between the gender and age composition of the under-studied COVID-19 patients. This further illustrates the necessity of thorough genomic surveillance with more completed demographic data to assess the emerging and circulating SARS-CoV-2 variants and prevalence of COVID-19 worldwide.

To study phylogenetic relationships, the subset data of globally circulating five VOCs sequences were analyzed in Nextstrain (Figure 1F). The molecular clock phylogeny revealed that (i) nearly all five VOCs emerged independently during late 2020 to late 2021, (ii) genome sequences of the same VOCs were more closely related, and (iii) the Omicron variant is the most commonly circulating variant currently found to have more than ~40 mutational differences relative to a reference strain, confirming that evolutionary pressures shaped the novel Omicron variant to distinguish itself from other variants. The evolutionary rate of five VOCs has been estimated to be 29.56 substitutions per year. Notably, pairwise comparison of a genome sequence of the Omicron variant (EPI_ISL_7547731) with other variants (Alpha EPI_ISL_7856427; Beta EPI_ISL_7814263; Gamma EPI_ISL_7846411; and Delta EPI_ISL_7861981) showed 95.6–96.1% genetic similarity. The Omicron variant shares 96% genome similarity with the reference strain. Interestingly, the reference strain shares a similar percent homology (though not identical changes) with bat coronavirus (bat/Yunnan/RaTG13/2013) [2], indicating the impact of genetic changes and structural variations in driving the evolution of SARS-CoV-2 variants.

### 3.2. Potential Effect of Omicron Mutations on Host Immune Response

**Figure 2:**
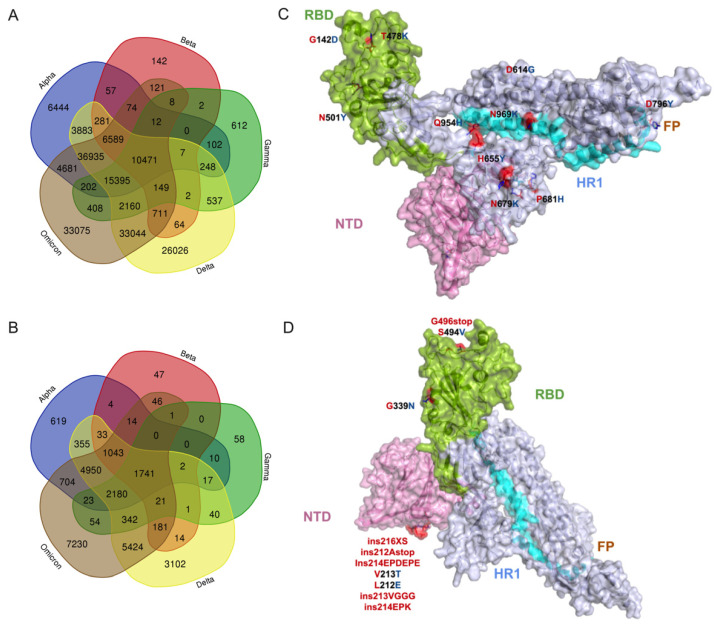
The common and exclusive mutations as well as structural changes in the proteome of Omicron variants. (**A**) Comparison of mutational profile data between 5 VOCs. In total, 12,002,213 sequences retrieved from GISAID’s EpiCoV database were used for this analysis. (**B**) Comparison of spike mutational profile data between 5 VOCs. (**C**) Illustration of the top 10 high-prevalence common spike glycoprotein mutations (D614G, frequency 99.50%; T478K, 83.28%; G142D, 73.15%; P681H, 60.31%; N501Y, 56.86%; H655Y, 51.69%; N679K, 50.54%; D796Y, 49.77%; N969K, 49.65%; Q954H, 49.54%) shared by 5 VOCs. (**D**) Illustration of the top 10 high-prevalence mutation and structural changes (G339N, 0.145%; ins216XS, 0.060%; ins212Astop; 0.373%; ins214EPDEPE, 0.021%; V213T, 0.020%; L212E, 0.020%; ins213VGGG, 0.016%; ins214EPK, 0.014%; G496stop, 0.010%; S494V, 0.010%) that are exclusive to Omicron variants on the monomer’s tertiary structure. Amino acid substitutions and structural changes in Omicron variants relative to the reference strain are represented in red stick models. Four domains in spike are highlighted as follows: (i) green for receptor binding domain (RBD), (ii) purple for N-terminal domain (NTD), (iii) brown for fusion peptide (FP) domain, and (iv) cyan for the heptad repeats-1 (HR1) domain. Grey is for inter-domain regions.

The comparison of the whole mutational profiles of proteome of five SARS-CoV-2 VOCs identified 33,075 signature mutations and structural changes exclusive to the Omicron variant (Figure 2A), in which 10,280 amino acid substitutions and structural changes were uniquely identified in the Omicron structural proteins (Figure 2B; Tables S2 and S3). These were not detected in the other four VOCs. If more than one amino acid or structural changes were identified in one position, then each change was considered individually for the analysis. We also identified that 74,359 mutational/structural changes commonly occurred in Omicron and Alpha variants, which is a larger number than common changes shared between Omicron and Beta (18,135) or Gamma (28,805) but a lesser number than those shared with Delta (105,454) (Figure 2A). All five VOCs shared a total of 10,471 common changes, suggesting these more evolutionarily conserved sites can be targeted for making novel disease control strategies. Among the 10,471 common changes, 1741 were identified in the spike protein (Figure 2A,B; Table S4), with three high-prevalence mutations (D614G, 99.50%; T478K, 83.28%; G142D, 73.15%) occurring in more than 70% of all VOC sequences studied (Figure 2C), whereas 7230 of 33,075 Omicron-specific signature changes were localized in the spike protein (Figure 2D; Tables S2 and S5). Remarkably, 1459 of 7230 changes identified in the spike were exclusively detected in the receptor binding domain (RBD) of the Omicron variant (Table S2). While all RBD mutations appear to be important to the behavior of SARS-CoV-2, identifying the precise pathobiological effects of these unique Omicron signature mutations, especially high-prevalence changes on ACE2 binding affinity, will facilitate our understanding of the variant’s rapid transmissibility, infectivity, and disease severity. A recent affinity and kinetics study reported that S477N and N501Y amino acid changes in other VOCs enhance transmission mainly by increasing binding to the ACE2 receptor, while the K417N mutation aids immune escape [25], suggesting that these changes in Omicron may also be involved in the same function. Nine single amino acid substitutions in the RBD (S373P, frequency 46.91%; K417N, 41.9%; N440K, 40.47%; G446S, 14.95%; S477N, 46.72%; T478K, 83.28%; G496S, 18.08%; Q498R, 45.62%; N501Y, 56.85%) (Table S4) were detected commonly within the binding site of the monoclonal antibodies, providing a potential reason for the observed loss of antibody binding or neutralization. However, further detailed studies are required to verify the impact of amino acid changes in the spike protein, particularly on RBD and available monoclonal antibody therapeutics [13].

A comparison of three other structural proteins—M, E, and N—among the five variants revealed that 194, 66, and 677 changes are commonly shared, respectively. However, in the Omicron variant, there were 1011 (M), 204 (E), and 1835 (N) changes exclusively identified in other three structural proteins (Tables S3 and S5). Similarly, analysis of the mutations in the non-structural proteins of Omicron showed 83,965 amino acid substitutions, 6877 deletions, and 7310 insertions in nsp1–16 polyproteins, whereas 8438 amino acid substitutions, 1902 insertion, and 585 deletions were found in the rest of the proteins ns3, ns6, ns7a, ns7b, and ns8 (Table S6). Overall, our analysis showed that, among the Omicron’s structural and non-structural proteins, the spike and polyproteins have undergone major genetic changes [26,27]. Moreover, further studies are required to verify the impact of these amino acid and structural changes in the currently circulating novel Omicron lineages, such as BA.4, BA.5, and BQ.1.

Further, we analyzed the influence of Omicron’s spike mutations on neuropilin 1 (NRP-1) host receptor binding. It is known that three amino acid changes in the furin cleavage site (H655Y, N679K, P681H) of the spike protein could aid viral transmission. For instance, the P681H mutation in the Alpha variant has been found to be involved in enhancing spike cleavage, resulting in increased viral transmission. Our analysis showed that these three mutations are identified in the Omicron variant, suggesting a possible increase in viral transmission. We also interpret that the proline-681 to histidine change in Omicron spike, resulting in a hydrophobic side chain substituted by a charged side chain, alters the interaction between the neuropilin 1 (NRP-1)-B1 domain with spike CendR peptide (furin cleavage site), similar to that observed in the Alpha variant [28]. This mutation shortens the distance between H681 residue and the interacting surface residues of NRP1 by 2 Å (reference with P681 = 16 Å; variant with H681 = 14 Å) [28], suggesting a strong interaction between the Omicron spike protein and NRP1, which may result in enhanced penetration of the virus into the central nervous system (CNS). Altogether, these amino acid changes in the spike protein reveal that infection by the Omicron variant is possibly enhanced by invasion of the CNS and potentially increased transmission compared to the early SARS-CoV-2 variant.

### 3.3. Molecular Docking Analysis of Omicron Proteins with Known Antiviral Drugs

**Figure 3:**
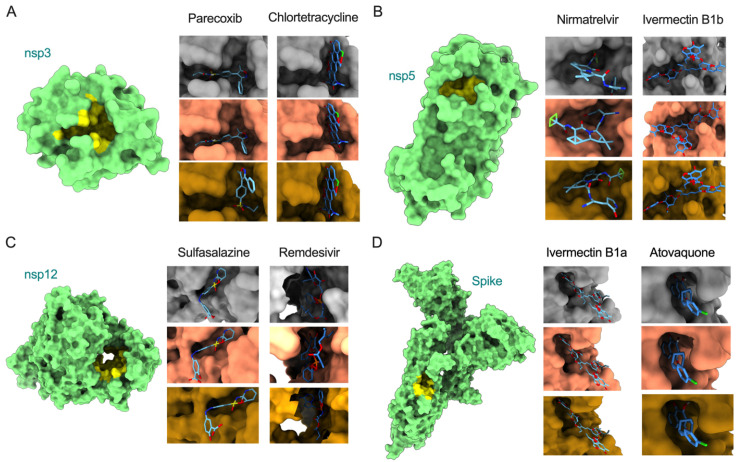
Surface model of binding of inhibitors to the active sites of nsp3, nsp5, nsp12, and spike (models with green color, left). Illustration of docking models (reference = grey; Delta = salmon; Omicron = goldenrod) for binding energy conformations are provided for (**A**) parecoxib and chlortetracycline bound to nsp3, (**B**) nirmatrelvir and ivermectin B1b bound to nsp5, (**C**) sulfasalazine and remdesivir bound to nsp12, and (**D**) ivermectin B1a and atovaquone bound to spike.

COVID-19 patient management can be improved by complementing current approved treatments with repurposing existing drugs as an imperative option. While the spike protein is a primary target for vaccine and neutralizing antibody-based therapeutic development, recent studies have demonstrated alternative promising antiviral targets, including nsp3 (papain-like protease), nsp5 (main protease), and nsp12 (RNA-dependent RNA polymerase, RdRp) [29,30,31]. Thus, we performed molecular docking analysis for eight antiviral drugs against these four target proteins (Spike, nsp3, nsp5, and nsp12) to identify if mutations in the Omicron variant alter the binding efficiency of the drugs. We also compared the overall patterns with respective proteins of reference and the Delta variant. The binding efficiency of inhibitors were characterized based on a scoring function output of AutoDock Vina v1.1.2 [20]. The ligands with the lowest binding affinity (lowest docking score) were considered potential inhibitors of SARS-CoV-2. The mutations in Omicron proteins that change drug binding affinity are illustrated in Figure 3, and the interacting residues and scoring outputs of the drugs are itemized in Table 1 and Figure S1.

Our analysis showed that remdesivir exhibited lower binding affinity with the Delta variant’s nsp12 (−7.4 kcal/mol delta; −6.4 kcal/mol reference strain) but exhibited higher binding affinity with Omicron’s nsp12 (binding energy: −8.0 kcal/mol) by interacting with fifteen residues, in which two (Cys622, Asn691) interact via hydrogen bonds (Figure 3 and Figure S1; Table 1). This suggests that the active site of the Omicron RdRp could be considered further to combat the COVID-19 pandemic. Similarly, nirmatrelvir showed a high binding affinity with Omicron’s nsp3 (−7.8 kcal/mol) by interacting with eleven residues, of which one (Glu166) formed a hydrogen bond. Interestingly, this drug exhibited similar binding affinity with the Delta variant and reference strain, suggesting that this drug may be an ideal candidate to treat multiple variants. However, upon widespread use of these drugs, there may be a possibility for emergence of drug-resistant viral strains. The binding affinities of all other six drugs (chlortetracycline, parecoxib, ivermectin B1b, sulfasalazine, ivermectin B1a, and atovaquone) were similar against each of the assessed viral proteins from the two variants and reference strain, suggesting nearly-conserved features of interacting residues from these proteins and the uniformity of these drugs in inhibiting SARS-CoV-2 strains. Only atovaquone uniformly interacted with the least number of residues (eight) of the spike protein (binding energy: −7.3 kcal/mol) across all three variants. However, no residues in the binding cavities interacted via hydrogen bonds. Notably, ivermectin B1a (−10 kcal/mol), ivermectin B1b (−10.2 to −9.8 kcal/mol), and sulfasalazine (−9.6 to −9.3 kcal/mol) exhibited high binding affinity with spike, nsp5, and nsp12 proteins, respectively. Altogether, the docking analysis revealed that: (i) the potential active cavities of the proteins studied are nearly conserved in different variants of SARS-CoV-2 and exhibit similar binding affinities, and (ii) remdesivir and nirmatrelvir may serve as active therapeutic drugs targeting the highly conserved nsp12 and nsp5 proteins, respectively, in the Omicron variant.

### 3.4. Impact of Key Amino Acid and Structural Changes in T-Cell Epitopes in Modulating the Host Cellular Immune Response

**Figure 4:**
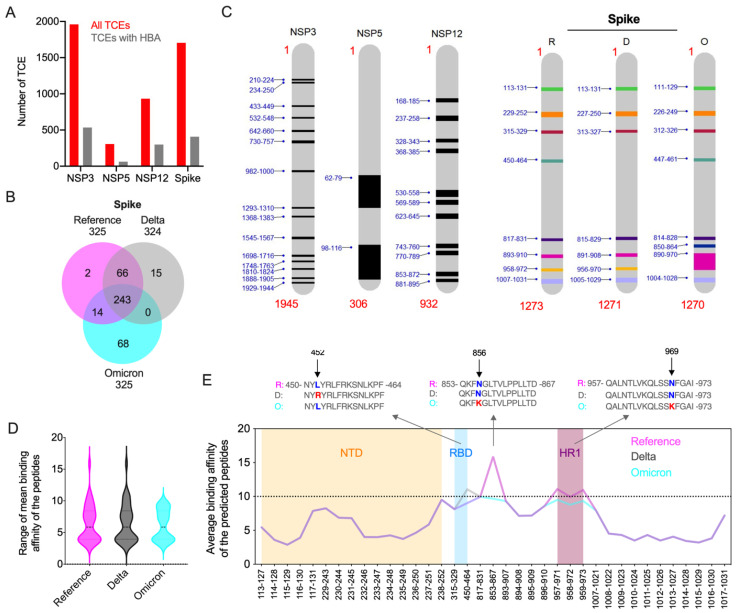
Prediction of high binding affinity CD4 TCEs of SARS-CoV-2 variants. (**A**) Overall distribution of HBA T-cell epitopes predicted from all nsp3, nsp5, nsp12, and spike proteins of three SARS-CoV-2 variants: reference, Delta, and Omicron. For each protein, the total number of predicted unique peptides (red) and the number of these peptides identified with HBA (grey) to at-least one prevalent global HLA allele is shown. (**B**) Comparison of high binding affinity TCEs from reference, Delta, and Omicron spike proteins. (**C**) The average binding affinity was calculated from the binding affinity score of each predicted fifteen amino acids peptides from four proteins with six prevalent HLA types studied. The functional hotspots in conserved nsp3, nsp5, and nsp12 proteins and spike are highlighted. Functional hotspots in spike predicted from reference (R) and two variants (D, Delta; O, Omicron) are shown. (**D**) The range and density of the median average binding affinity scores of the peptides predicted from the spike of the reference and two VOCs. For the comparison, the corresponding mean HBA TCEs in the reference and two variants (HBA score IC <= 10 nM or more in some peptides) are included. (**E**) The expanded version of panel D. The mean binding affinity score of each predicted peptide from the spike of three variants are plotted. Peptide coordinates provided in the *X*-axis are based on their locus in the reference protein. Mean HBA TCEs identified in NTD domain (orange), RBD domain (blue), and HR1 domain (plum) are highlighted. The key mutations that alter the binding affinity of the TCEs of Omicron and Delta variants are shown in the alignment of the predicted peptides.

Immunologically targeting SARS-CoV-2 proteins, especially the full-length spike protein and its RBD, using a vaccine can result in the generation of viral variants with immune-evasive mutations. The presentation of viral epitopes by class II MHC (HLA) isoforms plays a vital role in stimulating CD4^+^ Th1 and Th2 cell-mediated immune responses. As the binding affinity of HLA factors with SARS-CoV-2 CD4 epitopes determine host immune responses and the outcome of COVID-19, we analyzed the impact of Omicron’s mutations in modulating the host cellular immune response in silico. With respect to this immunity hypothesis, we predicted the peptides from the Omicron spike, nsp3, nsp5, and nsp12 proteins to identify the impact of these mutations on epitope binding affinity with the HLA alleles. Binding affinity of the peptides from these four proteins were compared with those of the Delta variant and a reference strain based on the predominant HLA alleles reported in the global population [2,18]. Complete analyses of peptides from four proteins of a reference strain and two variants showed that 20.5–32% of the predicted 15-mer peptides exhibited high binding affinity (HBA) to at least one of the global HLA types analyzed. Among these, most of the HBA T-cell epitopes (TCEs) were identified from nsp12 (32%) (Figure 4A). Because defining common epitopes from different variants is crucial for designing a universally potent subunit vaccine, we conducted a comparative assessment of HBA TCEs of the four proteins. Our analysis revealed that almost all HBA TCEs (63–529) of three non-structural proteins were commonly shared by the three strains assessed (Figure 4B and Figure S2). While these HBA TCEs of nsp3, nsp5, and nsp12 proteins are highly conserved among the reference and two VOCs, the spike displayed 2–68 HBA TCEs that are exclusive to reference and two VOCs (Figure 4B). Remarkably, the Omicron spike protein was predicted to contain a maximum of 68 HBA TCEs resulting from extensive novel mutations. The HBA T-cell epitopes identified in the functionally important regions of spike and non-structural proteins may critically modulate host immune responses to circulating SARS-CoV-2 variants. While many of the weak binding affinity regions in these proteins may favor viral evasion of host antiviral immunity, the HBA TCEs possibly induce protective immune response.

Since our preliminary epitope analyses identified the majority of HBA TCEs in the conserved nsp3, nsp5, and nsp12 proteins, we considered that these proteins may be more important in cell-mediated immune responses to SARS-CoV-2 infection than previously appreciated. Thus, we further analyzed the epitopes of these proteins along with the spike protein to understand the immunologically significant regions or functional hotspots within these proteins. Thus, to identify the functional hotspots in the four proteins that could potentially interact with predominant HLA alleles, the mean binding affinity was calculated for each of the predicted peptides of these proteins. We considered only CD4 TCEs with a high-confidence mean binding affinity score of IC ≤ 10 nM for further analysis. The data indicated 2–15 hotspots across the non-structural proteins from all three variants, in which the maximum number of hotspots were identified in nsp3 (Figure 4C). We identified 7–8 functional hotspots in the spike protein, in which the N-terminal domain (NTD; position aa 13-305) covered a maximum of 15 HBA TCEs (sliding window approach), while the receptor binding domain (RBD; aa 319-541) and heptad repeat region 1 (HR1; aa 912–984) secured the lowest number (aa 2-3) of HBA TCEs. Seven total HBA TCEs covering the Omicron spike domain spanning region from aa 890-970 (includes partial HR1) were identified to be a longer and immunologically important hotspot. Altogether, these findings indicate that added novel mutations in TCEs of the Omicron spike protein may have biological significance in SARS-CoV-2 immune evasion from T-cell response elicited by either natural infection or vaccination. While peptide regions in non-structural proteins may actively stimulate host immunity, much of the spike protein may favor host immune evasion.

Key amino acid changes in viral proteins have significant impact on epitope binding affinity, which changes viral behavior. With this hypothesis, we compared the epitopes with high mean HBA in four proteins across two variants and a reference strain. Since no amino acid changes in the mean HBA epitopes of non-structural proteins were observed, we focused on the highly mutated spike protein for further analysis. The range and density of the median average binding affinity scores of the peptides predicted from the spike protein of the three SARS-CoV-2 viruses showed that all mean HBA TCEs of Omicron exhibited high binding affinity (Figure 4D). For comparison, the corresponding mean HBA TCEs in the other strains (HBA score IC <= 10 nM or more in some peptides) were included. We identified two key mutations in the Omicron spike protein, namely hydrophilic/amphipathic changes N856K and N969K, leading to improved HBA for the TCEs carrying lysine (Figure 4E; Table S7), indicating that these changes may actively stimulate host immune response. While the former mutation was present in one HBA TCE (853–867), the latter was present in three HBA epitopes (957–971, 958–972, 959–973). The spike protein of the Delta strain had hydrophobic to hydrophilic amino acid change (L452R), which led to low binding affinity. In comparison, no amino acid changes were observed in the Omicron variant at this position relative to the reference strain, implying that these changes appear to favor the ability of the Delta variant to evade host immunity.

We also identified key structural changes in the Omicron spike protein, leading to decreased binding affinity (higher score), implying that these changes may facilitate viral evasion of host immunity. For instance, hydrophilic amino acid changes R19T in the Omicron spike protein (5-LVLLPLVSSQCVNLTTRTQL-24) relative to that of Delta, covering six 15 mer epitopes, led to reduced HBA binding affinity for the TCEs carrying threonine (average of mean HBA changes from 23.8 to 30.7). Similarly, two mutations in the Omicron spike protein, i.e., hydrophilic changes N348D and Q352H (Delta 936-LSSTASALGKLQ**N**VVN**Q**NAQALNTLV-961; Omicron 935-LSSTASALGKLQ**D**VVN**H**NAQALNTLV-960), also led to decreased HBA (average of mean HBA from 35.2 to 49.5) for the TCEs carrying aspartic acid and histidine. The combined amino acid changes G142D in the Omicron and Delta spike proteins compared to reference and three-amino-acid deletion 143-YYH-145 in the Omicron spike protein relative to Delta/reference (Delta 136-CNDPFLDVYYHKNNKSWMES-155; Omicron 134-CNDPFLD---HKNNKSWMESEFR-153) decreased the binding affinity enormously (38.3/46.1 to 69.9), indicating that these structural changes may help virus to escape from the host cellular immunity.

## 4. Conclusions

Our analysis based on about 12 million SARS-CoV-2 genomic sequences belonging to five VOCs showed that structural changes, including point mutations and indels occurring among different proteins, probably contribute significantly to SARS-CoV-2 genetic diversification and the generation of new variants. However, we discovered some immunologically important point mutations that are probably driven by selection by the human immune system, while the remaining mutations likely occurred due to adaptation to the host organism. The eight drugs we studied interacted with nearly-conserved residues in the binding cavities of four proteins from different SARS-CoV-2 variants, suggesting the potential of inhibitory action against the Omicron variant. Our exploration identified the eight functional hotspots in the three conserved non-structural proteins nsp3, nsp5, and nsp12, which were predicted to be functionally more important in immune responses to SARS-CoV-2 infections than previously appreciated, as the universal vaccine based on these conserved regions can effectively induce cellular immune responses to all the SARS-CoV-2 variants. Key structural changes in the Omicron spike protein at the epitope level may contribute to the wide range of immune evasion and high level of transmissibility of this novel SARS-CoV-2 variant among humans. Our analysis suggests that development of a novel vaccine may need to consider including epitopes from non-structural proteins and the 37 immunodominant CD4 epitopes of spike protein that we identified to induce an efficient T-cell response (Table S7). Further wet-lab experiments are required to confirm the immunological implications and the potential contribution of immunodominant epitopes in T-cell functional assays as well as their role in SARS-CoV-2 clearance from infected hosts. Together, our findings provide insights into the impact of novel mutations in the Omicron variant on drug interactions and CD4 epitope binding affinity to predominant HLA alleles. Our study also affirms the requirement for continuous global genomic surveillance of circulating SARS-CoV-2 variants, such as BA.4, BA.5, and BQ.1, to better understand the spread of emerging novel variants. As the virus evolves to become seasonally endemic, it is fundamentally important to continually evaluate the SARS-CoV-2 genomic surveillance and COVID-19-control strategies.

## Figures and Tables

**Table 1:** Molecular docking analysis of eight antiviral compounds (parecoxib, chlortetracycline, ivermectin B1a, ivermectin B1b, sulfasalazine, remdesivir, atovaquone, and nirmatrelvir) against their respective SARS-CoV-2 target proteins (nsp3, nsp5, nsp12, and spike). The 3D structure of four target proteins of the reference strain and the two variants Delta and Omicron were modeled before docking with inhibitors. The hydrogen bond-forming residues from the pool of interacting residues of each target protein are highlighted in bold. While we display all interacting residues from the reference strain, the interacting residues that were different from the reference are displayed for two variants for better readability. Refer to Figure S1 for visualization of inhibitors and target interactions.

Protein	Drug	Variant	Binding Affinity (kcal/mol)	Residues Involved in Interactions
nsp3	Parecoxib	Reference	−8.4	Ala242, Gly250, Gly251, Gly252, **Val253**, Ala333, Gly334, Ile335, Phe336, Ala358, **Phe360**, Leu364, Val539
		Delta	−8.4	Val359
		Omicron	−8.2	Asp226, Ile227, Ala256, Pro329, **Leu330**, Val359, Asp361
	Chlortetracycline	Reference	−8.5	Asp226, Ile227, Val228, Val253, Ala256, Pro329, **Leu330**, Ala333, Gly334, Val359, Phe360, Asp361, Leu364
		Delta	−8.5	No unique residues identified
		Omicron	−8.5	No unique residues identified
nsp5	Nirmatrelvir	Reference	−7.7	Phe140, Leu141, Asn142, Cys145, **His163**, Met165, Glu166, His172, Arg188, Gln189, Thr190, Gln192
		Delta	−7.6	His41, Met49, Leu167, Pro168, Asp187
		Omicron	−7.8	His41, Met49, Asp187
	Ivermectin B1b	Reference	−10.2	Thr26, **His41**, Ser46, Met49, Asn119, Gly143, Cys145, Met165, **Glu166**, Pro168, Arg188, Gln189, Thr190, Gln192
		Delta	−9.8	Thr24, Thr25, Asn142, Ala191
		Omicron	−10.2	No unique residues identified
nsp12	Sulfasalazine	Reference	−9.6	**Arg583**, Gly584, Gly597, **Ser592**, **His599**, **Asn600**, Met601, Lys603, **Thr604**
		Delta	−9.6	No unique residues identified
		Omicron	−9.3	No unique residues identified
	Remdesivir	Reference	−6.4	Arg553, Asp618, Tyr619, Pro620, **Lys621**, Cys622, Asp623, Asn691, Ser759, Asp760, Asp761, Glu811, Ser814
		Delta	−7.4	Tyr455, Arg624, Ser682, Thr687
		Omicron	−8	Tyr455, Arg533, Lys545, Arg555, Thr556, Trp617, Arg624, Ser682
Spike	Ivermectin B1a	Reference	−10	Lys811, Pro812, Ser813, Arg815, Asp820, Phe823, Gly832, Phe833, Ile834, Lys854, **Val860**, Thr866, Glu868
		Delta	−10	No unique residues identified
		Omicron	−10	No unique residues identified
	Atovaquone	Reference	−7.3	Thr732, Leu828, Ala831, Gly832, Phe833, Ile834, Val860, Pro862
		Delta	−7.3	No unique residues identified
		Omicron	−7.3	No unique residues identified

## Supplementary Materials

The following supporting information can be downloaded at: https://www.mdpi.com/article/10.3390/v14112461/s1, Figure S1: Interactions between inhibitors and target proteins of reference, Delta, and Omicron variants (column 1–3, respectively). The name of the target protein’s residues taking part in hydrophobic interactions are represented in black, whereas those taking part in hydrogen bonds are represented in green. Hydrophobic interactions are represented as red, continuous lines on a semicircle, whereas the hydrogen bonds are represented as a broken green line extending between the interacting atoms of residues and the inhibitor. (A) The interactions of nsp3 with parecoxib or chlortetracycline and nsp5 with nirmatrelvir or ivermectin B1b are shown. (B) The interactions of nsp12 with sulfasalazine or remdesivir and spike with ivermectin B1a or atovaquone are shown; Figure S2: Comparison of high binding affinity TCEs from reference, Delta, and Omicron nsp3, nsp5, and nsp12; Table S1: Virus nomenclature and metadata details of the sub-dataset of globally circulating SARS-CoV-2 belonging to five VOCs; Table S2: Common and unique mutations of spike protein among the VOC, with emphasis on Omicron variant. The nature of the mutations in each VOC are identified in comparison with a reference strain sequence (EPI_ISL_402124). This table summarizes common and unique mutations to their respective domains (NTD, N-terminal domain; RBD, receptor binding domain; HR1 and HR-2, heptad repeats-1 and -2; FP, fusion peptide; TM, transmembrane; CT, cytoplasmic tail) in the tertiary monomeric structure of the spike protein. Uncharacterized amino acids are represented as “X”. Insertions (prefix “ins”), deletions (suffix “del”), and point mutations are represented in this table by a single-letter abbreviation of amino acids followed by their position in the reference protein; Table S3: Common and unique mutations of envelope (E), membrane (M), and nucleocapsid (N) protein among the VOCs, with an emphasis to Omicron variant. The nature of the mutations in each VOC are identified in comparison with a reference strain sequence (EPI_ISL_402124). This table summarizes common and unique mutations to their respective domains in the tertiary monomeric structure of E, M, and N. Uncharacterized amino acids are represented as “X”. Insertions (prefix “ins”), deletions (suffix “del”), and point mutations are represented in the table by a single-letter abbreviation of amino acids followed by their position in the reference protein; Table S4: Mutations, insertions, and deletions that are commonly present in the structural proteins (spike, envelope, membrane, and nucleocapsid) of the five VOCs provided along with the frequency (%) of each change in 12,002,213 VOC sequences; Table S5: Mutations, insertions, and deletions that are exclusively present in the structural proteins (spike, envelope, membrane, and nucleocapsid) of the Omicron variant provided along with the frequency (%) of each change in 6,104,697 Omicron sequences; Table S6: Mutations found in various proteins (spike, envelope, membrane, nucleocapsid, nsp1–16, ns3, ns6, ns7a, ns7b, and ns8) of the Omicron variant. The mutations are identified in comparison with a reference strain sequence (EPI_ISL_402124). This table characterizes the mutations as insertions, deletions, or point mutations. All the mutations of structural proteins (spike, envelope, membrane, and nucleocapsid) are represented by a single-letter abbreviation of amino acids followed by their position in the reference. All the mutations of non-structural proteins (nsp1–16, ns3, ns6, ns7a, ns7b, and ns8) are represented by a prefix of the protein name followed by the single-letter abbreviation of amino acids and their position in the respective amino acid sequence of the reference; Table S7: Mean high binding affinity score of TCEs predicted from the spike of all three viral variants. The starting and ending positions of the epitopes in the spike, mean HBA score, and amino acid sequences of the epitopes are provided.
