# Supplementary material for: In Silico Genome Analysis Reveals the Evolution and Potential Impact of SARS-CoV-2 Omicron Structural Changes on Host Immune Evasion and Antiviral Therapeutics
Source: Viruses. 2022 Nov 6;14(11):2461. doi: 10.3390/v14112461 (PMC9697451; doi:10.3390/v14112461)
Supplement: Supplementary file 1 [file viruses-14-02461-s001.zip › Supplementary Figures.pdf]

(A) **(B)**

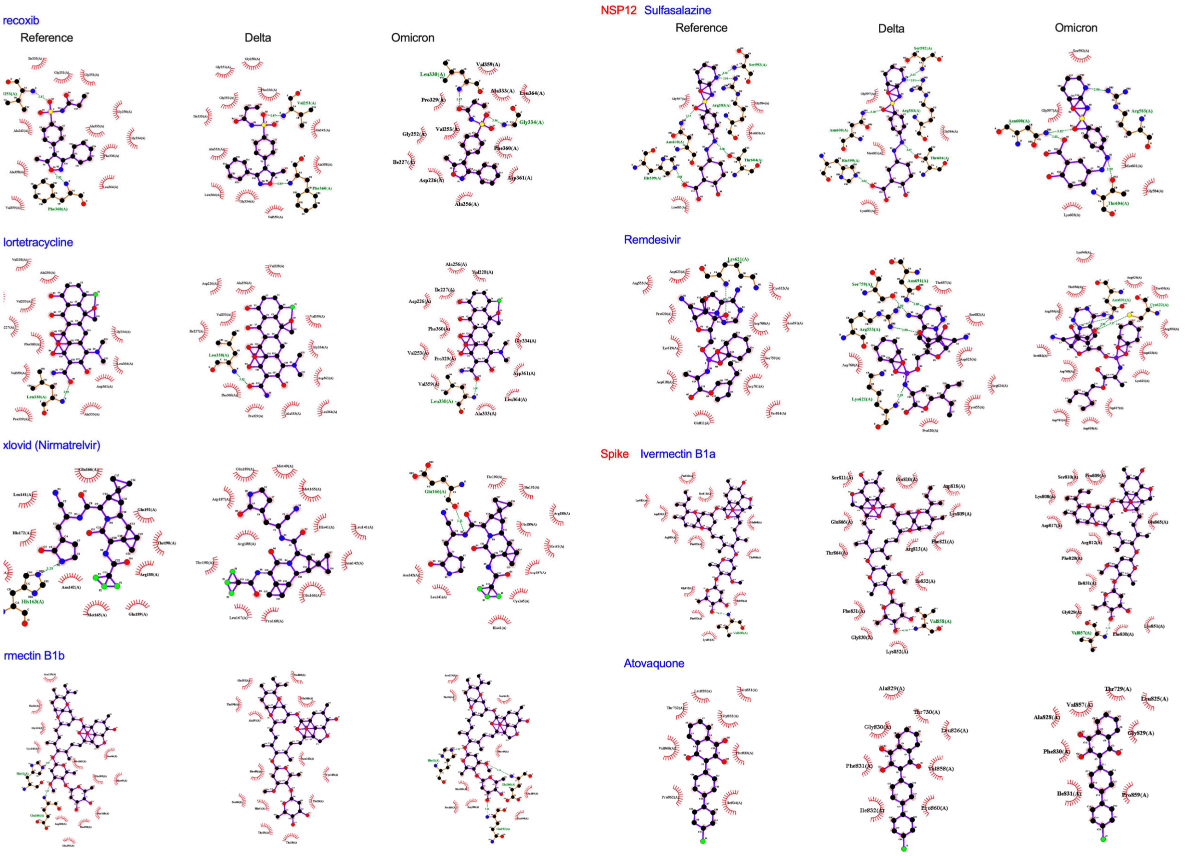

Figure S1. – Interactions between inhibitors and target proteins of Reference, and two variants Delta and Omicron (column 1-3, respectively). The name of the target protein's residues taking part in hydrophobic interactions are represented in black, whereas, those taking part in hydrogen bonds are represented in green. Hydrophobic interactions are represented as red continuous lines on a semicircle, whereas, the hydrogen bonds are represented as broken green line extending between the interacting atoms of residues and the inhibitor. (**A**) The interactions of nsp3 with Parecoxib or Chlortetracycline, and nsp5 with Nirmatrelvir or Ivermectin B1b are shown. (**B**) The interactions of nsp12 with Sulfasalazine or Remdesivir, and Spike with Ivermectin B1a or Atovaquone are shown.

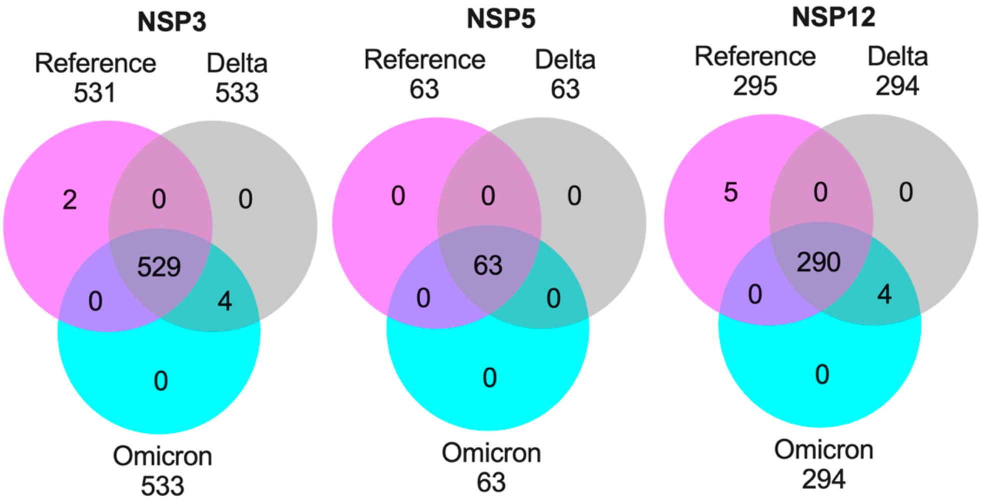

Figure S2. – Comparison of high binding affinity TCEs from Reference, Delta and Omicron nsp3, nsp5 and nsp12.
